# Social Determinants of Health and COVID-19 Among Patients in New York City

**DOI:** 10.21203/rs.3.rs-70959/v1

**Published:** 2020-09-15

**Authors:** Jennifer Woo Baidal, Amanda Y. Wang, Katarina Zumwalt, Dahsan Gary, Yael Greenberg, Ben Cormack, Stephanie Lovinsky-Desir, Kelsey Nichols, Neil Pasco, Andres Nieto, Jessica S. Ancker, Jeff Goldsmith, Dodi Meyer

**Affiliations:** Columbia University Irving Medical Center; Columbia University Vagelos College of Physicians & Surgeons; Columbia University Mailman School of Public Health; NewYork-Presbyterian; NewYork-Presbyterian; NewYork-Presbyterian; Columbia University Irving Medical Center; Columbia University Irving Medical Center; Columbia University Irving Medical Center; NewYork-Presbyterian; Weill Cornell Medicine; Columbia University Mailman School of Public Health; Columbia University Irving Medical Center

**Keywords:** Covid-19, racial/ethnic disparities, testing, housing

## Abstract

**Background:**

Covid-19 testing and disease outcomes according to demographic and neighborhood characteristics must be understood.

**Methods:**

Using aggregate administrative data from a multi-site academic healthcare system in New York from March 1 – May 14, 2020, we examined patient demographic and neighborhood characteristics according to Covid-19 testing and disease outcomes.

**Results:**

Among the 23,918 patients, higher proportions of those over 65 years old, male sex, Hispanic ethnicity, Medicare, or Medicaid insurance had positive tests, were hospitalized, or died than those with younger age, non-Hispanic ethnicity, or private insurance. Patients living in census tracts with more non-White individuals, Hispanic individuals, individuals in poverty, or housing crowding had higher proportions of Covid-19 positive tests, hospitalizations, and deaths than counterparts.

**Discussion:**

Variation exists in Covid-19 testing and disease outcomes according to patient and neighborhood characteristics. There is a need to monitor Covid-19 testing access and disease outcomes and resolve racist policies and practices.

## Introduction

New York experienced a surge in patients with Covid–19 in 2020, with 188,545 cases in New York City through May 14, 2020 [1]. Social determinants of health such as racism, income, and housing conditions play key upstream roles in etiologies of health and health disparities [2]. A recent ecological study showed variation in hospitalizations and deaths across New York City according to borough [3]. Variation in Covid–19 testing results and disease outcomes according to both individual and neighborhood characteristics has not been reported. In order to design approaches to contain Covid–19, the epidemiology of Covid–19 test results and disease outcomes must be understood.

Here, we describe patient demographics and neighborhood social determinants of health among consecutive patients who underwent Covid–19 testing at a multi-site health care system in New York. We also display the geographic distributions of outcomes.

## Methods

NewYork-Presbyterian (NYP) is the largest academic healthcare system in New York City, with more than 2 million encounters annually, and is affiliated with both Columbia University Vagelos College of Physicians and Surgeons and Weill Cornell Medicine. NYP encompasses 10 campuses located in New York City and Westchester. In this retrospective, observational cohort study, patients of any age who underwent a Covid–19 test at NYP from March 1, 2020 through May 14, 2020 were included if their residential address was geocoded to New York City. Results from nasal swab SARS-CoV–2 polymerase chain reaction (PCR) tests and administrative data were used to categorize outcomes. Covid–19 outcomes according to demographic characteristics, zip code, and census tract were obtained from existing aggregate administrative data across all NYP campuses. Analyses were deemed non-human subjects research by the authors’ Institutional Review Board. Covid–19 test results and disease outcomes were defined in discrete categories as negative test, positive test without hospitalization or known death (termed positive test), positive test with hospitalization but no known death (termed hospitalized), and positive test with known death (termed known death). Patients were represented once in the dataset. If a patient had multiple tests, the results of the most recent test were used.

Patient age, sex, race, ethnicity, insurance type according to each Covid–19 test and disease outcome were provided in aggregate from administrative data. Patients with both Medicaid and Medicare were included only in the Medicaid insurance category. Because data was provided in aggregate, counts of 1 were masked by providing a count of 1.5 for zip codes and census tracts to summarize those areas with a range of 1 to 2 individuals. Aggregate counts for zip code and census tract according to Covid–19 outcome were calculated as 0, 1.5, and then ordinally for 2. Neighborhood characteristics were calculated using 2018 American Community Survey data on race, ethnicity, poverty, and housing crowding in census tracts. Using descriptive statistics, we calculated census tract characteristics by quintiles according to Covid–19 outcomes. To align with publicly available maps of New York City which display data by zip code, zip codes were used to map the proportion of negative tests; proportion of New York City Covid–19 cases attributable to NYP patients [4]; proportion of NYP cases hospitalized without death; and death-to-case ratio to map using *ArcGIS* ArcMap (Version 10.6.1. Redlands, CA: Environmental Systems Research Institute, Inc., 2018). Zip codes with fewer than 50 tests performed at NYP were excluded from this visualization, as was Staten Island (only 1 zip code with 50 tests).

## Results

Overall, 24,461 patients with mean age 51.9 (SD 22.2) years and 57.5% female had a test performed at NYP and lived in New York City. Some patients had indeterminate test results, leaving a total 23,918 patients with confirmed test results included here.

Table 1 shows patient demographic characteristics according to Covid–19 test results and disease outcomes for those with confirmed test results. Mean age was lowest among those with negative tests and highest among those with known death. The majority of females had negative tests while males had higher proportions of positive tests, hospitalizations, and deaths. Hispanic patients tended to have a lower proportion of negative tests and higher proportion of hospitalizations and deaths than other racial/ethnic groups. Compared to those with private insurance or other payor type, patients with Medicare or Medicaid had lower proportions of negative tests and higher proportions of positive tests, hospitalizations, and known deaths.

**Figure 1:**
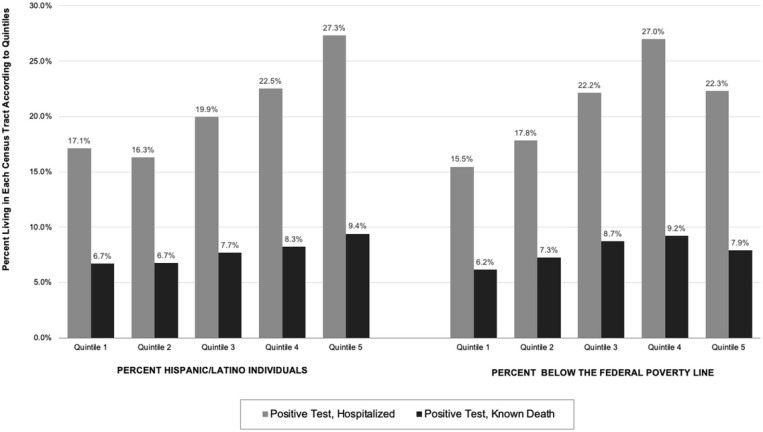
Neighborhood characteristics according to Covid-19 hospitalizations without death and known deaths. Data from 23,918 patients in New York City with Covid-19.

Table 1 and Figure 1 show neighborhood characteristics according to Covid–19 test results and disease outcomes. Those living in neighborhoods with fewer non-White individuals, fewer Hispanic/Latino individuals, fewer individuals living below the federal poverty line (FPL), or less housing crowding tended to have higher proportions of negative Covid–19 tests and lower proportions of hospitalizations and known deaths compared to those in neighborhoods with more non-White individuals, more Hispanic/Latino individuals, more individuals living below the FPL, and more housing crowding.

**Figure 2:**
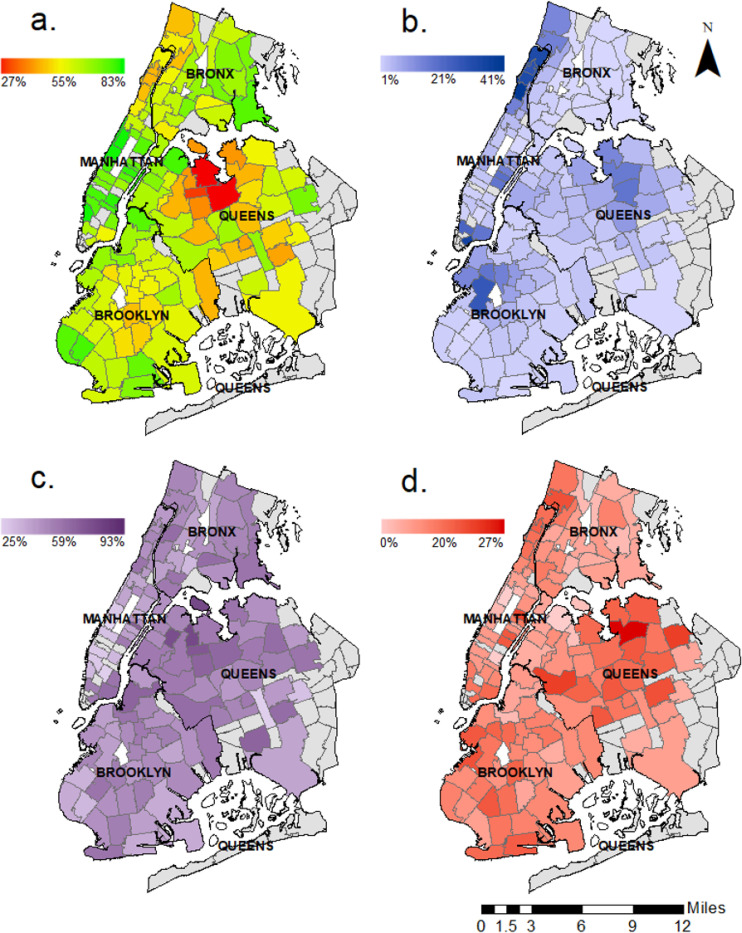
Covid-19 testing and disease outcomes by zip code. Data from 22,488 NewYork-Presbyterian (NYP) Patients living in New York City. Caption: Data from zip codes with fewer than 50 Covid-19 tests at NYP and Staten Island were excluded in all panels a. Proportion of negative tests per zip code b. Proportion of total cases attributable to NYP patients per zip code c. Case ratio of hospitalizations without known death per zip code d. Death-to-case ratio per zip code

Figure 2 shows maps of Covid–19 test results and disease outcomes among NYP patients in New York City. NYP patients made up a substantial proportion of New York City cases of Covid–19 in northern Manhattan, the lower tip of eastern Manhattan, and parts of the Bronx, Brooklyn, and Queens. Lower Manhattan, northern Bronx, and parts of Brooklyn had a high proportion of negative tests; these are all relatively affluent areas of the city. Some zip codes in Queens had the highest rates of Covid–19 positive tests, hospitalizations, and known deaths for NYP patients.

## Discussion

In this large sample of consecutive patients in a multi-site healthcare system in New York, differences in Covid–19 test results and disease outcomes according to age, sex, race/ethnicity, and insurance types were identified. Variation according to neighborhood-level social determinants of health and zip code existed. Those living in census tracts with more racial/ethnic minorities, individuals living in poverty, and more housing crowding had higher proportions of Covid–19 positive tests, hospitalizations, and known deaths than counterparts in other neighborhoods.

These results align with New York City data that show Hispanic/Latino individuals have the highest death rates from Covid–19, and that case rates are higher among those living in northern Manhattan and the outer boroughs than those in the more affluent areas of Manhattan [5]. These findings also suggest that those living in neighborhoods with more poverty and housing crowding—which are social determinants of health rooted in a history of structural racial and ethnic segregation and discrimination [6]—had higher proportions of positive tests, hospitalizations, and deaths. A higher proportion of negative tests were found in more affluent, predominantly White neighborhoods, which could signal greater testing accessibility and less severe disease in those neighborhoods compared to neighborhoods with greater poverty and more racial/ethnic minorities. Notably, however, as shown in Figure 1, the proportion hospitalized and died was slightly *lower* in the neighborhoods with the highest poverty than in neighborhoods with the next level of poverty, a disruption in the pattern which may reflect lack of access to healthcare rather than improved outcomes. Our results also align with findings in a recent study of critically ill patients at two NYP hospitals affiliated with Columbia University Irving Medical Center where 62% of patients admitted to intensive care units were Hispanic/Latino [7]. A series of 5,700 patients at another New York health care system with mostly White patients found that those with older age and male sex were among the majority of hospitalized patients [8]. Most existing Covid–19 literature has focused on hospitalizations and deaths. Little is known about those with negative tests or non-hospitalized patients. The current study adds to the literature by including a diverse patient population, those with negative tests, and those with Covid–19 who were not hospitalized.

This study has several limitations. The data were aggregate; thus individual-level statistical analysis was not possible and conclusions about causality cannot be drawn. This study is not representative of the entire city and not all census tracts and zip codes were equally represented.

In sum, among this cohort of over 20,000 patients in New York City, variation in Covid–19 test results and disease outcomes according to age, sex, race/ethnicity, insurance status, and neighborhood characteristics was identified. Widespread testing and contact tracing will be required to contain Covid–19 long-term. The findings support the need to monitor Covid–19 testing access and disease outcomes according to patient and neighborhood characteristics, resolve long-standing structural racial/ethnic discrimination and segregation, and address modifiable factors such as poverty and housing crowding in order to achieve health equity and reduce transmission of disease among the entire population.

## Figures and Tables

**Table 1.** Patient Demographics and Neighborhood Characteristics According to Covid-19 Test Res and Disease Outcomes. Data from 23,918 NYP Patients with Covid-19 Testing Results Between Ma 1, 2020 - May 14, 2020.

	Covid-19 Test Results and Disease Outcomes				
	Total tested with results available (N = 23,918)	Negative Test	Positive test, Not Hospitalized	Positive test, Hospitalized	Positive test, Known Death
**Patient Demographics**					
Age, mean (SD), years		46.4 (22.4)	49.2 (18.3)	60.7 (18.0)	74.8 (13.7)
Age, n (%), years					
0–21	1,483	1,290 (87.0)	105 (7.1)	86 (5.8)	2 (0.1)
22–45	8,484	6,288 (74.1)	1,191 (14.0)	949 (11.2)	56 (0.7)
46–65	6,620	3,327 (50.3)	1,048 (15.8)	1,907 (28.8)	338 (5.1)
>65	7,331	3,187 (43.5)	575 (7.8)	2,194 (29.9)	1,375 (18.8)
Sex, n (%)^a^					
Female	13,733	9,024 (65.7)	1,623 (11.8)	2,366 (17.2)	720 (5.2)
Male	10,181	5,064 (49.7)	1,296 (12.7)	2,770 (27.2)	1,051 (10.3)
Race/Ethnicity, n (%)^b^					
Hispanic	6,245	3,011 (48.2)	856 (13.7)	1,810 (29.0)	568 (9.1)
White, non-Hispanic	4,619	3,209 (69.5)	453 (9.8)	653 (14.1)	304 (6.6)
Black, non-Hispanic	3,481	1,974 (56.7)	540 (15.5)	720 (20.7)	247 (7.1)
Asian, non-Hispanic	1,793	1,136 (63.4)	155 (8.6)	346 (19.3)	156 (8.7)
Other, non-Hispanic	943	518 (54.9)	114 (12.1)	239 (25.3)	72 (7.6)
Unknown, non-Hispanic	474	295 (62.2)	63 (13.3)	86 (18.1)	30 (6.3)
Insurance type, n (%)^c^					
Medicaid^d^	7,677	4,209 (54.8)	744 (9.7)	1,977 (25.8)	747 (9.7)
Medicare	3,238	1,556 (48.1)	273 (8.4)	934 (28.8)	475 (14.7)
Private	6,551	4,311 (65.8)	1,031 (15.7)	950 (14.5)	259 (4.0)
Other	4,411	2,892 (65.6)	671 (15.2)	718 (16.3)	130 (3.0)
**Neighborhood Characteristics**					
Percent Non-White Individuals					
Quintile 1 (0% – 32.67%) - lowest	4,257	2,951 (69.3)	459 (10.8)	612 (14.4)	235 (5.5)
Quintile 2 (32.67% – 60.76%)	4,412	2,796 (63.4)	511 (11.6)	769 (17.4)	336 (7.6)
Quintile 3 (60.76% – 86.42%)	5,780	3,105 (53.7)	749 (13.0)	1,372 (23.7)	554 (9.6)
Quintile 4 (86.42% – 96.95%)	6,153	3,218 (52.3)	819 (13.3)	1,571 (25.5)	545 (8.9)
Quintile 5 (96.95% – 100%) - highest	3,710	1,991 (53.7)	530 (14.3)	917 (24.7)	272 (7.3)
Percent Hispanic/Latino Individuals					
Quintile 1 (0% – 7.39%) - lowest	3,674	2,367 (64.4)	431 (11.7)	630 (17.1)	246 (6.7)
Quintile 2 (7.39% – 14.08%)	4,417	2,810 (63.6)	588 (13.3)	721 (16.3)	298 (6.8)
Quintile 3 (14.08% – 24.12%)	4,333	2,595 (59.9)	541 (12.5)	864 (19.9)	333 (7.7)
Quintile 4 (24.12% – 47.6%)	4,661	2,691 (57.8)	534 (11.5)	1,051 (22.6)	385 (8.3)
Quintile 5 (47.6% – 100%) - highest	7,225	3,597 (49.8)	973 (13.5)	1,975 (27.3)	680 (9.4)
Percent Below Federal Poverty Threshold					
Quintile 1 (0% – 7.4%) - lowest	4,344	2,921 (67.3)	482 (11.1)	672 (15.5)	269 (6.2)
Quintile 2 (7.4% – 12.3%)	3,906	2,491 (62.4)	502 (12.6)	712 (17.8)	201 (7.3)
Quintile 3 (12.3% – 18.8%)	4,421	2,480 (56.1)	575 (13.0)	980 (22.2)	386 (8.7)
Quintile 4 (18.8% – 28.4%)	6,384	3,219 (50.4)	854 (13.4)	1,724 (27.0)	587 (9.2)
Quintile 5 (28.4% – 88.4%) - highest	5,167	2,949 (57.1)	655 (12.7)	1,153 (22.3)	410 (7.9)
Percent with Household Crowding					
Quintile 1 (0% – 3.1%) - lowest	3,599	2,390 (66.4)	429 (11.9)	549 (15.3)	231 (6.4)
Quintile 2 (3.1% – 6%)	4,969	3,215 (64.7)	583 (11.7)	846 (17.0)	325 (6.5)
Quintile 3 (6% – 9.4%)	4,380	2,517 (57.5)	570 (13.0)	919 (21.0)	374 (8.5)
Quintile 4 (9.4% – 14.6%)	5,453	3,067 (56.3)	688 (12.6)	1,256 (23.0)	442 (8.1)
Quintile 5 (14.6% – 100%) - highest	5,913	2,872 (48.6)	799 (13.5)	1,671 (28.3)	571 (9.7)

a Excludes 4 patients with missing data

b Includes 17,555 patients with self-reported race or ethnicity

c Includes 21,877 patients with insurance information

d Includes Medicaid/Medicare dual-eligible recipients

Abbreviations: NYP = NewYork-Presbyterian
